# Antinociceptive potentiation of pethidine (demerol) by clomipramine in the late phase of formalin test in mice

**Published:** 2012-06-10

**Authors:** Hellen N Kariuki, Titus I Kanui, Paul G Kioy

**Affiliations:** 1 Department of Medical Physiology, University of Nairobi, P. O. Box 30197 (00100) Nairobi, Kenya; 2 Department of Veterinary Anatomy and Physiology, University of Nairobi, P. O. Box 30197 (00100), Nairobi, Kenya

**Keywords:** Pethidine, clomipramine, formalin test, late phase, potentiation, mice

## Abstract

**Background:**

Pethidine, an opioid analgesic is used for pain management. Clomipramine a tricyclic antidepressant primarily used for mood management is also used to treat pain. The objective of this study was to investigate the potentiation of the analgesic effects of sub-threshold dose of pethidine by a tricyclic antidepressant, clomipramine.

**Methods:**

The antinociceptive activities of clomipramine and pethidine alone and in combination were investigated in Swiss albino mice using the formalin test. Normal saline was employed as the control. Ten animals were used in each experiment.

**Results:**

Pethidine 5mg / kg failed to cause any significant effect while the 6.25, 7.5, 8.75 and 10.0mg /kg showed highly significant antinociceptive effect (p< 0.01) compared to the controls in the late phase of formalin test. Clomipramine 0.5 mg / kg did not show any significant effect while 0.75 mg / kg caused a significant effect (p< 0.05) while 1.00 and 1.25mg /kg caused a very highly significant antinociceptive effect (p< 0.001) in the late phase of formalin test compared to the vehicle treated animals. The combination of pethidine 5mg / kg and clomipramine 0.75mg / kg caused a highly significant antinociceptive effect (P<0.01) in the late phase of formalin test.

**Conclusion:**

This study demonstrates a marked reduction in the time spent in pain behaviour produced by the combination of low dose pethidine and clomipramine in the late phase of formalin test. The findings demonstrate the potentiation of a narcotic analgesic by a tricyclic antidepressant.

## Background

Antidepressants are widely used in the management of pain conditions and syndromes [1–3]. They have also been found to alleviate pain in the depressed and the non-depressed patients [1, 2, 4]. They provide a decrease in the perception of pain in both animals and humans [1, 2].

The Tricyclic antidepressants (TCAs) are used primarily in the clinical treatment of mood disorders such as major depressive disorder, as well as chronic pain, neuralgia or neuropathic pain, and, headache or migraine [5]. The TCAs show efficacy in the clinical treatment of a number of different types of chronic pain, notably neuralgia or neuropathic pain and fibromyalgia. The precise mechanism of action in explanation of their analgesic efficacy is unclear, but it is thought that they indirectly modulate the opioid system in the brain downstream via serotonergic and noradrenergic neuromodulation, among other properties. They are also effective in migraine prophylaxis, though not in the instant relief of an acute migraine attack. They are also effective to prevent chronic tension headaches [5–8].

In experimental studies, Tricyclic antidepressants have been used in pain management and produced antinociception in man [9], in arthritic rats [10], in the tail flick test and in the inflammatory pain model in rats [11].

Clomipramine (Anafranil) a tricyclic antidepressant (TCA) was developed in the 1960s by the Swiss drug manufacturer Geigy (now known as Novartis) and has been in clinical use worldwide ever since. Clomipramine has been shown to be effective in treating patients with central pain [12]. It has been shown to potentiate the antinociceptive effects of morphine in the carageenin test in rats [11].

Pethidine is a fast-acting opioid analgesic drug and was the first synthetic opioid. It is indicated for the treatment of moderate to severe pain, and is delivered as a hydrochloride salt in tablets, as a syrup, or by intramuscular, subcutaneous or intravenous injection. It has also been used for pre medication during surgery and post operation pain management. For much of the 20th century, pethidine was the opioid of choice for many physicians and it has been prescribed for acute pain and for chronic severe pain [13].

The aim of this study was to investigate the potentiation of the analgesic effects of sub-threshold dose of pethidine (Demerol) by a tricyclic antidepressant, clomipramine.

## Methods

### Animals

Adult Swiss albino mice of both sexes weighing 20–26 g were used. The animals were kept under normal laboratory conditions of light, temperature and humidity and allowed access to food and water ad libitum for at least seven days, before the commencement of the experiments [14]. Institutional approval was sought and granted by the Department of Medical Physiology and the School of Medicine, University of Nairobi and. The “Principle of Laboratory Animal Care” [14] guidelines and procedures were also followed in this study [14]. All the tests were carried out in the daytime and in a quiet laboratory setting with ambient illumination and temperature similar to those of the animal house. Animals were allowed to acclimatize to the test laboratory setting an hour before the experiments commence. The animals were randomly divided into groups of 10 per each dosage of drug and vehicle.

### Standard drugs

The reference drugs used were: Clomipramine (Novartis), Batch number 19F0075, Pethidine (Demerol) hydrochloride (AstraZeneca Pty Ltd), Batch number 20020510 and Formalin (Sigma), Batch number 031140.

### Administration

Standard drugs and vehicle (normal saline) were injected 40 microlitres intraperitoneally (i. p.) using a 17 gauge needle thirty minutes prior to the formalin test. Clomipramine dosages used were; 0.25, 0.50, 0.75, 1.00, and 1.25mg/kg, while pethidine (Demerol) hydrochloride the dosages were 5.0, 6.25, 7.5, 8.75 and 10 mg/kg. In the combined drugs experiment the dosage used were pethidine (Demerol) hydrochloride (5mg/kg) and clomipramine (0.75mg/kg). Sensorimotor test To evaluate possible nonspecific muscle relaxant or sedative effects of the drugs, animals were tested on an apparatus that consisted of three rods, diameter 2.5 cm, with the height of 20, 32, and 64 cm. Animals were placed on top of each rod for 20 seconds to test their sensorimotor function. The animals were selected 24 h previously by eliminating those mice which did not remain or had no firm grip on the rods for two consecutive periods of 60 s. Animals were injected with the drugs one hour before the test. Control animals received the same volume of vehicle (0.9% NaCl) solution 40 microlitre i. p. one hour before being tested. The cut-off time used was 20s.

### Formalin- induced licking

Swiss albino mice (20–26 g) were treated with Clomipramine (0.25, 0.50, 0.75, 1.00, and 1.25 mg/kg), pethidine (Demerol) hydrochloride (5.0, 6.25, 7.5, 8.75 and 10 mg/kg) and in the combined drugs experiment pethidine (Demerol) hydrochloride (5 mg/kg) and clomipramine (0.75 mg/kg) one hour before formalin injection. The procedure was similar to that described previously [15, 16]. Using a microliter syringe and a 26 gauge needle, 20µl of 1% formalin in 0.9% Normal saline was injected subcutaneously into the dorsal side of the right hind paw of each animal. The animal was returned to the observation chamber immediately after injection and the observation period started. The amount of time (in seconds) the animal spends in paw elevations, rapid paw shakes, flinches, biting and /or licking of the injected paw were recorded using a stop watch in five minutes blocks for a period of sixty minutes. The animals’ behaviour was also recorded in terms of whether it was quiet, active or asleep during the period of the experiment. The amount of time spent licking the injected paw was scored. Ten animals were used in each experiment.

### Statistical analysis

Cumulative data in both phases of formalin test scores (early 1-10min. and late 15-60min) following formalin injection were pooled and analysis was done using one-way ANOVA followed by Shaffes post-hoc test. The differences in the test- versus control (vehicle treated) values were considered to be statistically significant at P < 0.05. Data is expressed as mean ± S.E.M (standard error of the mean). The dose was the independent variable.

## Results

### Sensorimotor test

The clomipramine (0.25, 0.50, 0.75, 1.00, and 1.25mg /kg), pethidine (Demerol) hydrochloride (5.0, 6.25, 7.5, 8.75 and 10mg /kg) and in the combined drugs experiment pethidine (Demerol) hydrochloride (5mg / kg) and clomipramine (0.75mg / kg) given one hour before sensorimotor testing, did not affect the motor performance of animals when compared with the control group response.

### Formalin test

**Figure 1:**
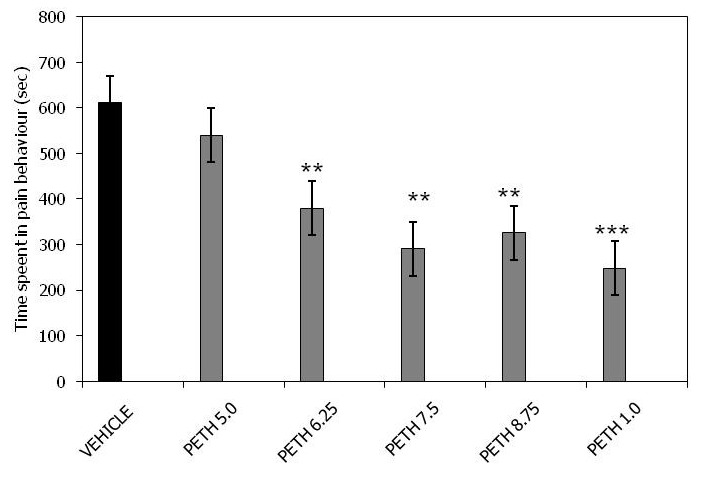
Pethidine late phase of formalin test. Each group represents the mean ± SEM of 10 animals. **p<0.01, ***p<0.001 when compared with the control value subsequent to ANOVA

Figure 1 presents the data from intraperitoneal injection of pethidine in the late phase of formalin test. Pethidine 5mg / kg failed to cause any significant effect (540.0 ± 57.6) compared to the controls (611.5 ± 66.7) while the 6.25 mg / kg (379.8 ± 32.6), 7.5 mg / kg (291.7 ± 33.1) and 8.75 mg / kg (326.1 ± 44.8) doses showed a highly significant antinociceptive effect on the time spent in pain behaviour (p< 0.01). The 10.0mg /kg (248.6 ± 58.7) dose showed a very highly significant reduction (p < 0.001) in the time spent in pain behaviour compared to the vehicle treated animals (611.5 ± 66.7).

**Figure 2:**
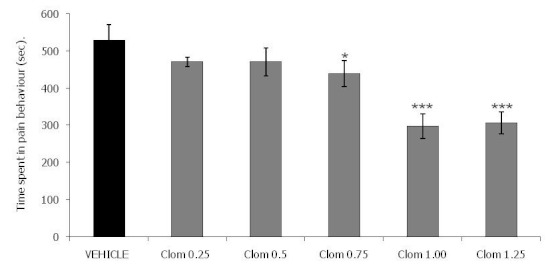
Clomipramine late phase of formalin test. Each group represents the mean ± SEM of 10 animals. *p<0.05, ***p<0.001 when compared with the control value subsequent to ANOVA

Figure 2 shows the results of intraperitoneal injection of clomipramine in the late phase of formalin test. Clomipramine 0.5 mg / kg (471.1 ± 12.4) dose did not show any significant effect in the late phase of formalin test compared to the vehicle treated animals (529.7 ± 40.9). The 0.75 mg / kg (439.4 ± 34.8) caused a significant effect (p < 0.05) on the time spent in pain behaviour. Clompramine 1.00 mg / kg (298.2 ± 33.3) and 1.25mg / kg (306.5 ± 29.0) caused a very highly significant antinociceptive effect (p< 0.001) in the time spent in pain behaviour in the late phase of formalin test compared to the vehicle treated animals (529.7 ± 40.9).

**Figure 3:**
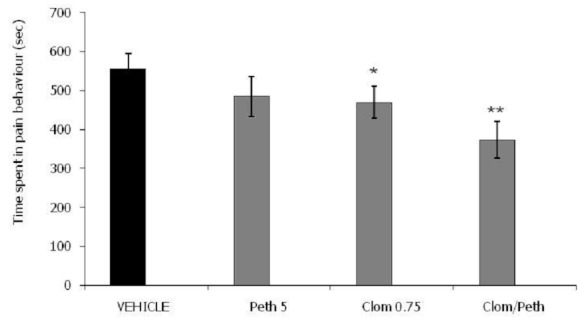
Combination of pethidine and clomipramine in the late phase of formalin test. Each group represents the mean ± SEM of 10 animals. *p<0.05, **p<0.01 when compared with the control value subsequent to ANOVA

Figure 3 illustrates the potentiation of pethidine by clomipramine after intraperitoneal injection of the drugs in the late phase of formalin test. The combination of pethidine (Demerol) 5 mg/kg and clomipramine 0.75mg / kg (373.3 ± 47.9) caused a highly significant reduction (P < 0.01) in the time spent in pain behaviour compared to the vehicle treated animals (555.4 ± 40.0) in the late phase of formalin test.

## Discussion

Formalin test is widely used as a tonic pain model to assay antinociceptive activity of chemical compounds [17]. It is generally accepted that centrally acting analgesics have effects on both phases whereas peripherally acting analgesics will affect only the first phase [18, 19]. This is because the injection of formalin results in the release of various neurotransmitters including glutamate and aspartate in the dorsal horn [20]. Therefore the early phase of the formalin test represents the transmission of nociceptive impulses while second or late phase of the formalin test represents the events of central sensitization and wind-up phenomena [17, 21].

In this study the early phase of formalin test did not show any significant difference between the combination of pethidine and clomipramine and the when each drug was used alone.

The analgesic effects of different pethidine (Demerol) dosages on the late phase of formalin test were evaluated and the 5mg /kg dose did not show any significant effect compared to the controls (P > 0.05). The pethidine (Demerol) dosages of 6.25, mg / kg and above significantly reduced the amount of time spent in pain behaviour in the formalin test.

Clomipramine 0.25mg / kg failed to cause any significant difference on the time spent in pain behaviour in the late phase of formalin test compared to the vehicle treated animals. Significant reduction in the time spent in pain behaviour (P < 0.05) in the late phase of formalin test was achieved following the administration of 0.75mg / kg dose and above.

The combination of clomipramine 0.75 mg / kg and the sub threshold parental dose of pethidine (Demerol) 5mg / kg showed a significant reduction (P < 0.01) on the time spent in pain behaviour in the late phase of formalin test compared to the vehicle treated animals. Clomipramine potentiated the sub threshold dose of pethidine (Demerol) and the analgesic effects of the combination were more potent than each drug dose used alone. Similar results have been reported with amytriptiline and morphine in the tail flick test [22].

## Conclusion

In conclusion, this study demonstrates a significant antinociception produced by the combination of low dose pethidine (Demerol) and clomipramine in the late phase of formalin test. The findings demonstrate the potentiation of a narcotic analgesic by a tricyclic antidepressant.
